# Cognition, fear, and falls: psychological predictors of balance impairment in community-dwelling older adults

**DOI:** 10.3389/fpsyt.2025.1610894

**Published:** 2025-06-03

**Authors:** Batool Abdulelah Alkhamis, Basant Hamdy Elrefaey, Mohammad A. Almohiza, Khalid A. Alahmari, Mastour Saeed Alshahrani, Hani Hassan Alnakhli, Ghada Koura, Debjani Mukherjee, Saleh M. Kardm, Faisal M. Alyazedi, Ravi Shankar Reddy

**Affiliations:** ^1^ Program of Physical Therapy, Department of Medical Rehabilitation Sciences, College of Applied Medical Sciences, King Khalid University, Abha, Saudi Arabia; ^2^ Department of Health Rehabilitation Sciences, College of Applied Medical Sciences, King Saud University, Riyadh, Saudi Arabia; ^3^ Department of Surgery, College of Medicine, Najran University, Najran, Saudi Arabia; ^4^ Physical Therapy Department, Prince Sultan Military College of Health Sciences, Dahran, Saudi Arabia

**Keywords:** cognitive function, fear of falling, older adult, physical therapy, fall prevention, balance performance

## Abstract

**Objective:**

This study aimed to (1) assess the associations among cognitive function, fear of falling, and balance in community-dwelling older adults; (2) identify key predictors of balance performance; and (3) examine implications for fall prevention strategies.

**Methods:**

Eighty-four older adults (≥65 years) residing independently in the community were recruited during outpatient visits. Cognitive function was assessed using the Saint Louis University Mental Status (SLUMS), fear of falling was measured with the Falls Efficacy Scale-International (FES-I), and balance performance was evaluated using the Berg Balance Scale (BBS). Physical activity, demographic factors, and fall history were also recorded.

**Results:**

Bivariate correlations showed that higher cognitive function was associated with better balance (r = 0.45, p = 0.014), while greater fear of falling was linked to poorer balance (r = -0.52, p = 0.003). A hierarchical regression model revealed that cognitive function (β = 0.32, p = 0.002) and fear of falling (β = -0.44, p < 0.001) were significant predictors of balance performance, even after controlling for age, gender, physical activity, and fall history. Exploratory logistic regression showed that fear of falling (OR = 1.12, p = 0.002) and balance performance (OR = 0.91, p = 0.008) were significant predictors of fall history.

**Conclusion:**

Cognitive function and fear of falling are independent and meaningful predictors of balance performance in older adults. These findings support the integration of cognitive and psychological assessments into exercise-based fall prevention strategies.

## Background

Maintaining balance is essential for functional independence and fall prevention in older adults (1). Among community-dwelling older adult individuals, balance impairments are common and represent a major public health concern due to their strong association with increased fall risk, injury, reduced mobility, and loss of independence (2). Falls remain one of the leading causes of morbidity and mortality among older populations worldwide, with significant implications for healthcare systems and caregivers alike (3, 4).

While traditional fall prevention strategies have emphasized physical factors—such as muscle strength, joint stability, and vestibular function—emerging evidence highlights the vital role of non-physical factors, particularly cognitive function and psychological status, in postural control and fall risk (5, 6). Cognitive processes, including executive function, attention, and processing speed, are intricately linked with motor planning and adaptive responses to environmental challenges (7). Age-related cognitive decline can impair these processes, reducing an individual’s ability to maintain balance or respond appropriately to unexpected perturbations (8).

Simultaneously, fear of falling (FoF) has gained recognition as a modifiable psychological factor that significantly affects mobility and stability, even in individuals without a history of falls (9). FoF may lead to activity restriction, reduced physical fitness, maladaptive postural strategies, and heightened anxiety—all of which increase the likelihood of actual falls and contribute to a vicious cycle of fear, inactivity, and decline (10). Several theoretical frameworks support these associations. The cognitive-motor interference theory posits that when attention is divided between cognitive and motor tasks, performance in one or both domains may decline, which is particularly relevant in older adults who often struggle with dual-task balance activities (11). Additionally, neuroimaging studies have shown that prefrontal cortical regions involved in executive function are also activated during postural control, suggesting shared cognitive resources (12). The fear-avoidance model further explains how elevated fear of falling leads to self-imposed activity restrictions, deconditioning, and maladaptive postural behaviors that heighten fall risk (13). This integrative view underscores the need to assess psychological and cognitive domains alongside physical capabilities when addressing balance impairments in older adults (14).

Despite growing interest in these factors, research that integrates cognitive function and fear of falling as concurrent predictors of balance performance remains limited, particularly in non-Western, community-dwelling older adult populations (15). Although cognitive and psychological contributors to balance impairments have received growing attention in recent research, many fall prevention programs in clinical and community settings continue to emphasize physical training alone, with limited integration of these interconnected factors into routine practice (16). This study addresses this gap by examining a sample of independently living older adults in the southwestern region of Saudi Arabia. This context is underrepresented in global fall prevention research.

This study seeks to address this gap by examining the independent and combined associations between cognitive function, fear of falling, and balance performance in community-dwelling older adults. By doing so, we aim to inform more holistic, multidimensional fall prevention strategies that go beyond conventional physical rehabilitation. Specifically, this study aims to: 1) Assess the relationship between cognitive function, fear of falling, and balance performance. 2) Identify the key predictors of balance performance. 3) Explore the implications of these findings for designing integrated physical therapy interventions to mitigate fall risk.

## Materials and methods

### Ethical data and study design

This prospective cross-sectional study was conducted between April 22, 2023, and March 13, 2024, at the Department of Geriatrics and Physical Medicine, a tertiary care hospital specializing in geriatrics and rehabilitation. Ethical approval was obtained from the Institutional Review Board (IRB) of King Khalid University (Approval No. REC# 345-2-23) on March 23, 2023. All participants signed written informed consent prior to enrollment.

### Sample size estimation

*A priori* sample size calculation was performed using G*Power 3.1.9.7 for multiple linear regression with two primary predictors [cognitive function and fear of falling, as per Umegaki et al. (17)], a medium effect size (f² = 0.15), α = 0.05, and power = 0.80. The required minimum sample was 68 participants. Our final sample of 84 exceeded this threshold, ensuring sufficient statistical power for the planned analyses.

#### Participants

The participants in this study were community-dwelling older adults aged 65 years and older who attended outpatient services at the Department of Geriatrics and Physical Medicine at King Khalid University, KSA. For the purposes of this study, “community-dwelling” was defined as individuals who reside independently in their own homes, with no reliance on institutional care or assisted living facilities. All participants were ambulatory and functionally independent, attending outpatient services for routine health and mobility assessments. The sample consisted of community-dwelling older adults from the southwestern region of Saudi Arabia, predominantly of Arab ethnicity. This Middle Eastern context is characterized by traditional multigenerational living arrangements, limited institutional care, and culturally specific patterns of physical activity, fall risk perception, and healthcare access—factors that distinguish it from Western populations. The eligibility criteria included individuals who were independently mobile, had no history of major neurological or musculoskeletal disorders that could affect balance, and possessed sufficient cognitive capacity to understand and complete the study assessments. Participants diagnosed with advanced dementia, Parkinson’s disease, stroke, or other conditions that significantly impair motor control or cognitive function were excluded. Additionally, those with severe visual or auditory impairments that could interfere with balance and cognitive tests were also not eligible for the study. Participants were selected through a consecutive sampling method during routine outpatient visits. The selection process involved an initial screening by geriatric specialists, who assessed participants’ medical histories, physical conditions, and cognitive function to ensure they met the inclusion criteria. Fall history was assessed by asking participants whether they had experienced one or more falls in the previous 12 months. A total of 96 eligible individuals were approached, of whom 84 consented and completed the study assessments, yielding a response rate of approximately 87.5%.

### Physical activity assessment

Physical activity was self-reported using a structured format based on the Global Physical Activity Questionnaire (GPAQ). Participants were asked to indicate the number of days per week and the average duration per day they engaged in walking, moderate-intensity activities (e.g., household chores, brisk walking), and vigorous-intensity activities (e.g., heavy lifting, fast cycling). MET scores were then calculated using standard MET values: 3.3 for walking, 4.0 for moderate activity, and 8.0 for vigorous activity. The total physical activity level was expressed as MET-minutes per week.

#### Assessment of cognitive function using the SLUMS exam

Cognitive function was evaluated using the Saint Louis University Mental Status (SLUMS) Exam. This validated tool assesses a broad range of cognitive domains, including memory, attention, executive function, and logical reasoning (17). The SLUMS consists of 11 items with a total score ranging from 0 to 30, similar to SLUMS (18). However, SLUMS includes more executive function tasks and is considered more sensitive for detecting mild cognitive impairment (MCI), especially in community settings (18). Interpretation is education-dependent: scores of 27–30 (normal), 21–26 (MCI), and 1–20 (dementia) for individuals with a high school education (18). Trained clinicians administered SLUMS in quiet, standardized outpatient sessions. The SLUMS has demonstrated good internal consistency (Cronbach’s alpha = 0.82) and strong concurrent validity with the MMSE, particularly in detecting mild cognitive impairment in community-dwelling older adults (19).

#### Falls efficacy scale-international

Fear of falling was measured using the Falls Efficacy Scale-International (FES-I), a validated self-report questionnaire designed to assess individuals’ apprehension about falling during daily activities (20). Comprising 16 items, the FES-I covers various indoor and outdoor tasks such as household chores, stair navigation, and walking on uneven terrain (21). Participants rate their level of concern regarding falling while performing each activity on a scale of 1 to 4, where 1 indicates minimal concern and 4 indicates significant concern. Scores on the FES-I range from 16 to 64, with higher scores indicating heightened fear of falling. A score closer to 64 suggests that the individual experiences significant anxiety related to falls, which may impact their daily functioning and willingness to engage in physical activity. Conversely, lower scores indicate more confidence in performing daily activities without the fear of falling. Participants were given the FES-I questionnaire in a quiet, comfortable environment to encourage honest and thoughtful responses. They were instructed to complete the questionnaire independently, but assistance was provided if they had difficulty understanding any of the items or required clarification. For participants with visual impairments or literacy difficulties, a trained interviewer read the questions aloud and recorded their responses. The FES-I has shown high internal consistency (Cronbach’s alpha = 0.96) and test-retest reliability (ICC = 0.96), as well as established construct validity across diverse older adult populations (22).

#### Berg balance scale

Balance performance, the primary outcome in this study, was assessed using the BBS, a widely used and validated tool for evaluating functional balance ability in older adult individuals (23). The BBS consists of 14 tasks that assess both static and dynamic balance under various conditions (24). These tasks include standing unsupported, sitting to standing, transferring between chairs, standing on one foot, reaching forward while standing, turning 360 degrees, and standing with feet together, among others (24). Each task challenges different aspects of balance, such as postural control, weight shifting, and the ability to maintain stability during functional movements. Each element of the BBS is assessed using a scale ranging from 0 to 4 (24). A score of 0 indicates inability to perform the task, while 4 signifies independent completion with full control. The cumulative BBS score spans from 0 to 56, where higher scores reflect better balance and decreased likelihood of falling. Individuals scoring below 45 are typically deemed to be at heightened risk of falls. The assessments were conducted by trained physical therapists in a quiet, well-lit room free of distractions. The participants were provided clear verbal instructions before each task, and demonstrations were performed when necessary to ensure understanding. The physical therapists closely monitored the participants’ performance throughout each task, and safety precautions, such as having a chair or support nearby, were taken to prevent falls during the assessment. Each task was scored immediately after completion to ensure accurate recording. The therapists followed a standardized protocol to ensure consistency in scoring across all participants, and regular inter-rater reliability checks were performed to minimize scoring discrepancies.

Additional covariates included age, gender, and physical activity level, which were obtained through participant self-reports and confirmed through medical records. Physical activity was quantified using the Metabolic Equivalent of Task (METs), which estimates energy expenditure based on participants reported daily activities. Finally, the presence of chronic conditions and the number of medications were recorded from medical histories, as these factors could influence balance and cognitive performance. All data were collected and entered into a secure database for subsequent analysis. The BBS has been extensively validated, with excellent inter-rater (ICC = 0.98) and intra-rater reliability (ICC = 0.97), and strong predictive validity for identifying fall risk in older adults (25).

Quality control was maintained through several strategies. All assessments were conducted by trained clinicians using standardized instructions and protocols. Inter-rater reliability was ensured for the BBS by regular calibration sessions among assessors. Scores were recorded immediately after each task to minimize recall errors. Additionally, all data entries were independently verified by a second researcher to ensure accuracy and consistency in the dataset.

#### Data analysis

Data were analyzed using SPSS version 24. Parametric tests were used after confirming the normal distribution of key variables. Descriptive statistics (means and standard deviations) were calculated for cognitive function, fear of falling, and balance performance. Bivariate relationships were examined using Pearson’s correlation. Hierarchical multiple regression was conducted to identify predictors of balance performance (BBS). In Step 1, participant-level variables (age, gender [dummy coded], and physical activity) were entered. In Step 2, cognitive function (SLUMS score) was added, followed by fear of falling (FES-I score) in Step 3. This allowed for evaluation of incremental variance explained (ΔR²) at each stage. All standard regression assumptions—including normality, linearity, homoscedasticity, and multicollinearity—were tested and met. Statistical significance was set at a p-value of less than 0.05.

## Results

**Table 1** summarizes the demographic and clinical profiles of the participants. The study included older adults, with an average age in the early seventies, and a fairly even distribution between males and females. On average, participants exhibited a healthy body mass index and moderate cognitive function scores, as indicated by the SLUMS. The mean SLUMS score of 23.82 falls within the range indicative of mild cognitive disorder for older adults with at least a high school education, based on standard interpretation guidelines. This level of cognitive function is consistent with previously reported averages among community-dwelling older adults in the region. Fear of falling and balance performance varied across the group, with a notable proportion of participants having a history of falls. The mean FES-I score of 22.67 indicates a moderate level of concern about falling, based on established scoring thresholds where scores of 16–19 reflect low concern, 20–27 moderate concern, and 28–64 high concern. This suggests that, on average, participants were somewhat cautious about falling but not severely limited by fear. Additionally, the sample displayed moderate physical activity levels, and on average, participants reported taking several medications and having multiple chronic conditions.

**Table 1:** Demographic and clinical characteristics of the participants (n=84).

Variable	Mean ± SD or n (%)
Age (years)	72.54 ± 6.32
Gender (Male/Female)	36 (42.9%)/48 (57.1%)
BMI (kg/m²)	25.74 ± 3.18
Cognitive Function Score (SLUMS)	23.82 ± 3.10
Fear of Falling Score (FES-I)	22.67 ± 5.31
Balance Performance (BBS)	46.82 ± 7.12
History of Falls in the Past 12 Months (Yes/No)	40 (47.6%)/44 (52.4%)
Physical Activity Level (METs)	2568.34 ± 721.45
Medications (mean number)	4.25 ± 1.23
Chronic Conditions (mean number)	2.35 ± 0.87

METs, Metabolic Equivalent of Task; BMI, Body Mass Index.

**Figure 1:**
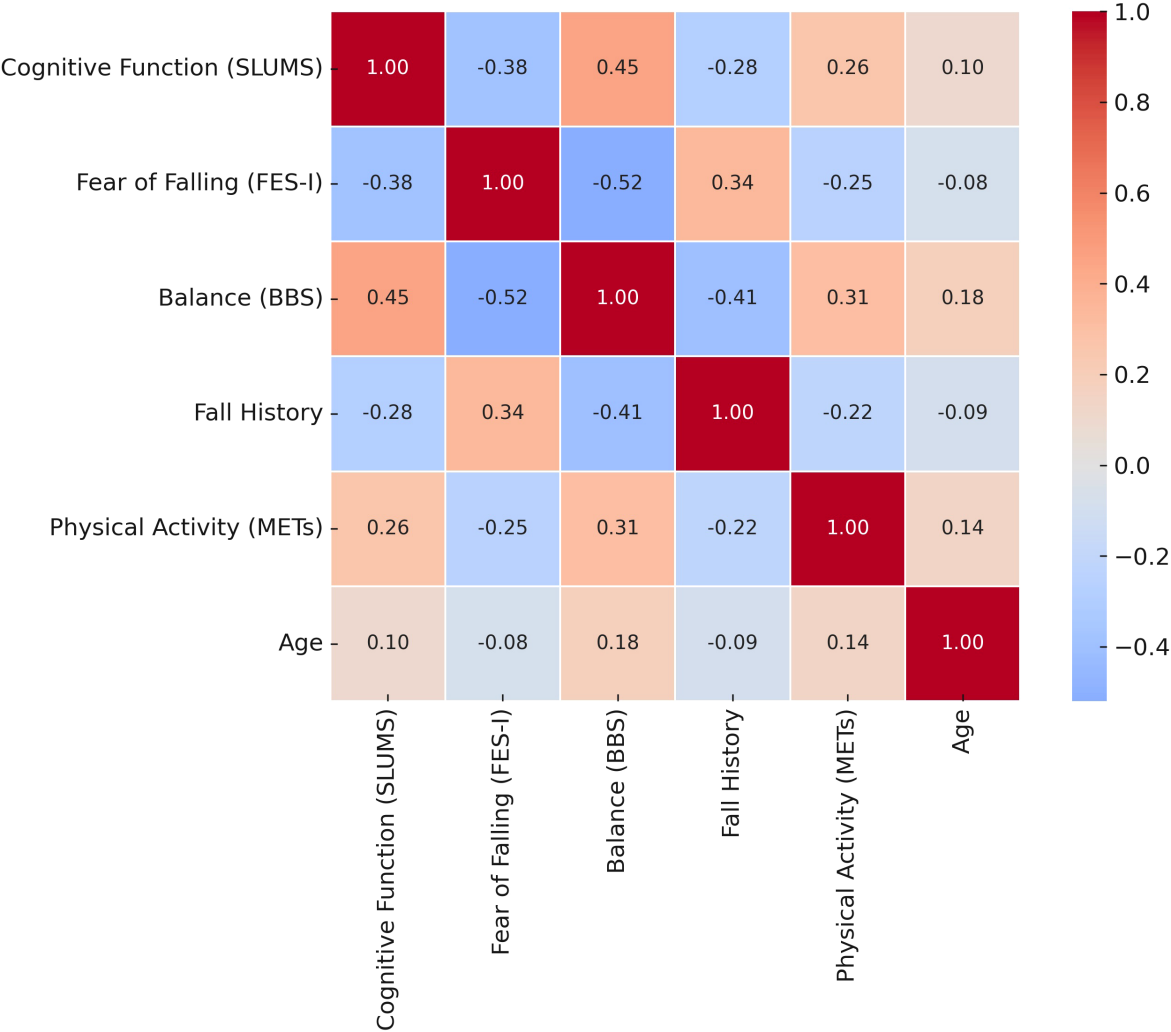
Heat map of bivariate correlations among cognitive, psychological, demographic, and functional variables.

Bivariate associations among all variables—including cognitive function, fear of falling, balance performance, fall history, age, gender, and physical activity—are presented in **Figure 1**. The multiple linear regression analysis identified both cognitive function and fear of falling as significant predictors of balance performance (**Table 2**). Cognitive function had a positive effect on balance performance, with a standardized coefficient (β) of 0.35 (p = 0.012), indicating that better cognitive function was associated with improved balance. In contrast, fear of falling negatively impacted balance performance, with a larger standardized coefficient (β = -0.47, p = 0.002), suggesting that greater fear of falling led to poorer balance.

**Table 2:** Multiple linear regression: cognitive function and fear of falling as predictors of balance performance.

Predictor	β	B	SE	t-value	95% Confidence Interval	p-value
Cognitive Function	0.35	0.42	0.11	3.82	0.14 to 0.56	0.012
Fear of Falling	-0.47	-0.61	0.14	-4.36	-0.67 to -0.27	0.002

β, standardized regression coefficient; B, unstandardized coefficient; SE, standard error; tl t-statistic; p-value, probability value.

A hierarchical regression was conducted to evaluate whether cognitive function and fear of falling predicted balance performance beyond self-reported fall history (**Table 3**). Fall history (Yes = 1, No = 0) was entered in Step 1, followed by cognitive function in Step 2, and fear of falling in Step 3. Fall history alone significantly predicted balance performance (β = -0.28, p = 0.011). Adding cognitive function and fear of falling significantly improved the model (ΔR² = 0.10, p = 0.006 and ΔR² = 0.11, p = 0.002, respectively), with the final model explaining 49% of the variance in BBS scores (R² = 0.49). Fear of falling emerged as the strongest predictor, followed by cognitive function. Additionally, a binary logistic regression using falls history as the outcome showed that fear of falling (OR = 1.12, p = 0.002) and balance performance (OR = 0.91, p = 0.008) were significant predictors, while cognitive function was not (p = 0.097). These findings highlight the predictive value of psychological and functional measures for both balance and fall risk. This logistic model was exploratory and aimed to assess whether cognitive, psychological, and functional variables could retrospectively predict fall occurrence as a binary outcome.

**Table 3:** Hierarchical regression predicting balance performance with demographics, cognitive function, and fear of falling.

Model Step	Predictor	β	B	SE	t	p-value	ΔR²	Total R²
Step 1	Age	-0.18	-0.24	0.11	-2.18	0.032		0.17
	Gender (Male = 0, Female = 1)	-0.09	-1.37	0.92	-1.49	0.141		
	Physical Activity	0.21	0.003	0.001	2.45	0.017	0.17	
Step 2	Cognitive Function	0.32	0.38	0.12	3.17	0.002	0.12	0.29
Step 3	Fear of Falling	-0.44	-0.54	0.13	-4.15	<0.001	0.15	0.44

β, standardized regression coefficient; B, unstandardized coefficient; SE, standard error; t, t-statistic; p-value, probability value; ΔR², change in R-squared; R², coefficient of determination.

**Figure 2:**
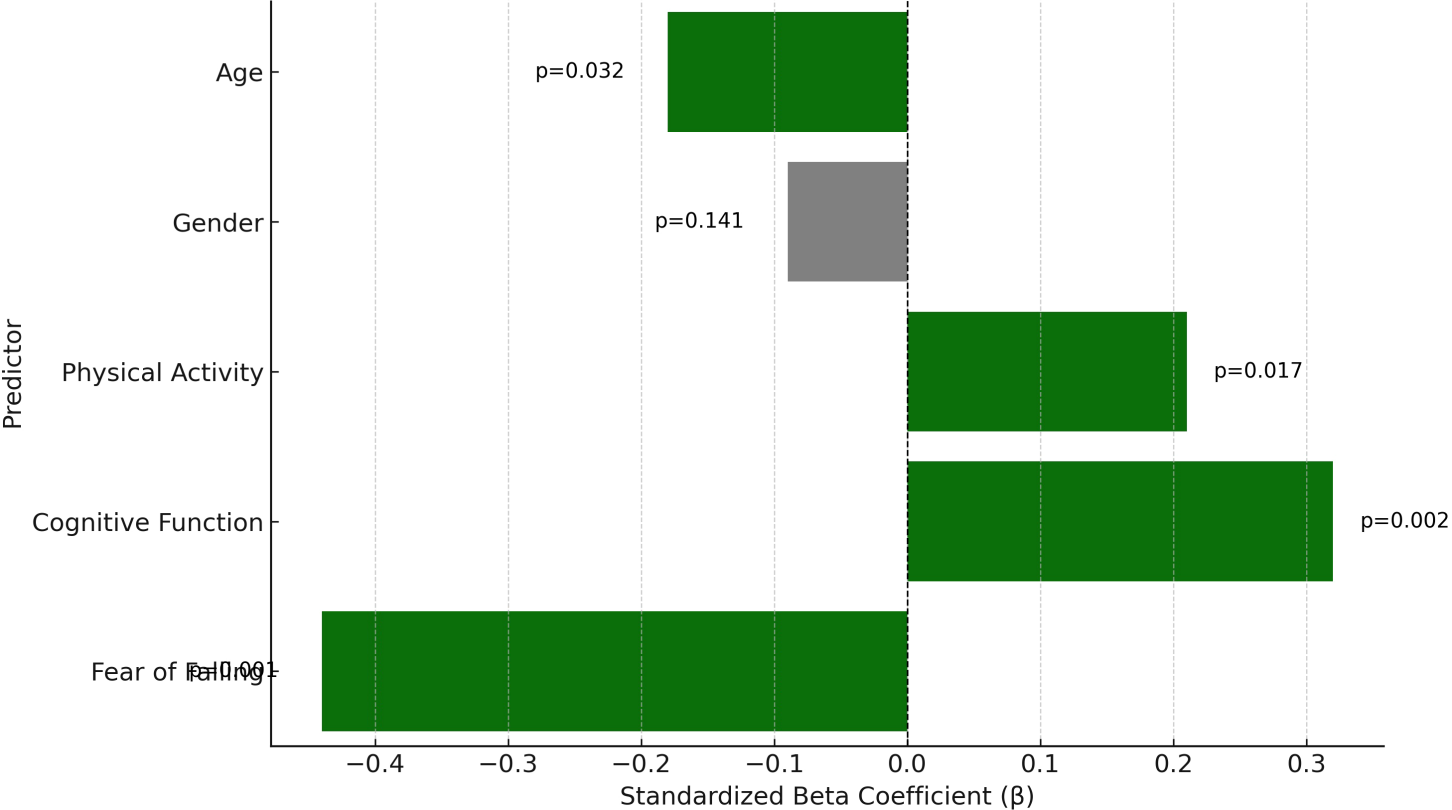
Standardized beta coefficients from hierarchical regression predicting balance performance (BBS).

The hierarchical regression analysis showed that age, cognitive function, and fear of falling were all significant continuous predictors of balance performance, with fear of falling demonstrating the strongest unique contribution (**Figure 2**). Gender did not emerge as a statistically significant predictor. These findings reinforce the relevance of cognitive and psychological factors beyond demographic or physical activity variables in explaining balance variability among older adults.

## Discussion

This study investigated the relationship between cognitive function, fear of falling, and balance performance in community-dwelling older adults. Our findings reveal that both cognitive function and fear of falling are significant, independent predictors of balance performance, even after adjusting for age, gender, and physical activity levels. These results align with a growing body of evidence indicating that balance control in older adults is not solely governed by physical capacity but is also significantly influenced by cognitive processes such as executive function, attention, and dual-task performance, as well as psychological factors like fear of falling and perceived control over movement (26–29).

The observed positive correlation between cognitive function and balance performance aligns with previous studies suggesting that executive functions—such as attention shifting, dual-tasking, and motor planning—play a critical role in maintaining postural control (26, 27). Impaired cognition may hinder the ability to process environmental cues or anticipate and correct imbalances, thereby increasing fall risk (30, 31). Our findings reinforce the necessity of incorporating cognitive assessments into fall risk evaluations, particularly in older adults who may not present with overt physical impairments.

Fear of falling emerged as the strongest predictor of impaired balance, even surpassing cognitive function. This highlights the profound impact of psychological factors on motor performance. 21,22 Elevated fear levels can lead to activity avoidance, reduced physical conditioning, and hypervigilant postural strategies that paradoxically increase the risk of falls (28, 29). These results support models such as the fear-avoidance cycle, which explain how anxiety-related behaviors contribute to functional decline (29). Moreover, our *post-hoc* analysis revealed significant group differences in balance based on levels of fear and cognition, underscoring the clinical relevance of these constructs.

The inclusion of physical activity, estimated from self-reported GPAQ data and converted into MET minutes per week using standard WHO metabolic equivalents, provides important context in modeling balance performance. Even after controlling for physical activity levels, both cognitive function and fear of falling remain significant predictors. This suggests that interventions focusing solely on physical fitness may be insufficient for improving balance in older adults [32, 33]. These findings support the need for multidimensional fall prevention strategies that combine cognitive training and psychological support with traditional exercise-based rehabilitation. Beyond muscle strengthening and gait training, interventions should incorporate cognitive rehabilitation, such as dual-task and executive function exercises [34, 35], as well as psychological approaches like cognitive behavioral therapy to address the fear of falling [6, 36]. Such integrated approaches may be especially effective in community settings, where early intervention can prevent functional decline [37].

Although balance performance was the primary outcome in this study, the clinical outcome of most significant importance —falls —was not directly measured over time. While we recorded self-reported fall history, the study design did not permit prospective fall tracking. Future research should incorporate longitudinal designs to assess whether cognitive function and fear of falling predict actual fall events, offering stronger clinical relevance and translational value.

A notable strength of this study is its focus on a community-dwelling sample of older adults from a Middle Eastern context—an underrepresented population in geriatric research. The use of well-validated assessment tools (SLUMS, FES-I, BBS) and robust statistical modeling further enhance the credibility of the findings.

### Limitations of The study

The cross-sectional design precludes conclusions about causality. Longitudinal or interventional studies are necessary to investigate whether improving cognitive function or reducing the fear of falling leads to measurable improvements in balance. The study also excluded individuals with severe cognitive impairments or those from institutionalized populations, which limits the generalizability of the findings. Additionally, other relevant variables such as sensory impairments or environmental hazards were not assessed and may interact with the primary predictors. Furthermore, the study did not account for environmental factors such as indoor layout, lighting, flooring surfaces, or climatic conditions, which may influence balance and fall risk, particularly in older adults with sensory or mobility impairments. Despite these limitations, our results contribute valuable insights into the interplay of cognitive, psychological, and physical factors in fall risk among older adults. The findings support the incorporation of cognitive screening and fear of falling assessments into standard geriatric evaluations and point toward more comprehensive, personalized intervention strategies.

## Conclusion

In conclusion, this study identified cognitive function and fear of falling as significant and independent predictors of balance performance in community-dwelling older adult individuals. Fear of falling had the most substantial impact on balance, followed by cognitive function, highlighting the critical role of both psychological and cognitive factors in maintaining postural stability. The findings underscore the importance of incorporating cognitive and psychological assessments, particularly targeting fear of falling, into exercise-based rehabilitation strategies. A multidimensional approach that integrates cognitive training and psychological support alongside traditional physical therapy may be more effective in improving balance and reducing fall risk in older adults. By adopting a multidimensional approach that addresses both cognitive and psychological aspects, healthcare professionals can improve mobility and overall quality of life in older adult populations at risk for falls.

## Data Availability

The datasets generated and analyzed during this study are available in the publicly accessible Zenodo repository. The data can be accessed via the following DOI: https://doi.org/10.5281/zenodo.13907449. Additional data or supporting materials can be made available from the corresponding author upon reasonable request, subject to institutional guidelines and participant confidentiality policies.
